# Delirium screening tools: A protocol for a systematic review and meta-analysis

**DOI:** 10.1097/MD.0000000000021595

**Published:** 2020-08-28

**Authors:** YuJuan Liu, Qian Zhang, Yayun Zhao, Zhuying Gao, Zhengyong Wei, Ziqi Guo, Meixi Chen, Qing Zhang, Xuemei Yang

**Affiliations:** a Second Hospital of Lanzhou University; b School of Nursing, Lanzhou University, Lanzhou, China.

**Keywords:** delirium, mechanically ventilated protocol, screening, systematic review

## Abstract

**Background:**

Delirium is a frequent form of acute brain dysfunction in mechanically ventilated patients. Screening tools have been developed to identify delirium, but it is unclear which tool is the most accurate. Therefore, we provide a protocol of systematic evaluation to assess the accuracy of delirium screening tools in mechanically ventilated patients.

**Methods:**

PubMed, PsycINFO, EMBASE, and the Cochrane Library will be searched. Studies involving mechanically ventilated patients which compared diagnostic tools with the Diagnostic and Statistical Manual of Mental Disorders criteria as a reference standard will be included. We will use MetaDiSC and STATA 15.1 to analyze carefully when a network meta-analysis is allowed.

**Results:**

This study will provide a high-quality synthesis to assess the accuracy of different screening methods in mechanically ventilated patients.

**Conclusion:**

The conclusion of our systematic review will provide evidence to judge which screening method is the best for mechanically ventilated patients.

## 1 Introduction

Delirium is a syndrome in mental fluctuating with an acute change in cognition, level of consciousness, and a decline in focus.^[1,2]^ Patients affected delirium intended to have poor outcomes, including longer hospital stays,^[3]^ a higher rate of hospital-acquired complications, and increased mortality.^[4,5]^ Mechanically ventilated patients with severe delirium encounter the continuous reduction of quality of life after discharge.^[6,7]^ The prevalence of delirium in mechanically ventilated patients is as high as 60% to 80%.^[8,9]^ In the United States, 1-year healthcare costs associated with delirium are estimated to be $38 billion.^[10]^ More than 8 of 10 mechanically ventilated adult patients had delirium,^[11]^ yet the consequences of delirium have been long underestimated.^[12]^ Because mechanical ventilation patients suffer from tracheal intubation and physical restraint, assessment of delirium is still a challenge in the mechanically ventilated patients.^[13]^

Although guidelines for the management of delirium have recommended that detection should be performed as early as possible,^[14]^ it was only rarely done because delirium monitoring was often complicated and time-consuming.^[15]^ Besides, the healthcare professional's ability to recognize delirium is poor, with around 50% of cases of delirium going unrecognized.^[16]^ In England, just 25% of intensivists routinely screen for delirium.^[17]^ Clinical practice guidelines recommend that the availability of a valid tool for delirium assessment is a crucial component in the detection of delirium,^[14]^ which can prompt more accurate diagnosis and avoid the adverse effects of undiagnosed and untreated delirium.^[18]^

Currently, indicators for delirium screening and diagnosis have not been uniformly recognized.^[19,20]^ Different screening tools have a variety of sensitivities and specificities. The time needed to complete the assessments also adds to the complexity of delirium detection.^[21]^ Different guidelines provide different recommendations. National Institute for Health and Care Excellence (NICE)^[22]^, the confusion assessment method for the intensive care unit (CAM-ICU) shall be used in the recovery room after surgery or critical care. The Scottish Intercollegiate Guidelines Network (SIGN) ^[23]^ recommends that in the emergency department, the 4AT (Arousal, Attention, Abbreviated Mental Test 4, Acute change) tool should be used for identifying delirium. Although many assessment tools are already in use, what assessment tools are most effective in mechanical ventilation patients remains unknown.

So far, several analyses have been conducted to determine which is the best for delirium screening in the ICU. Gusmao-Flores et al^[24]^ analyzed 11 studies and found that CAM-ICU and Intensive Care Delirium Screening Checklist (ICDSC) can be applied for the diagnosis of delirium in critically ill patients. But few meta-analysis focused on patients with mechanical ventilation. Because cognitive testing is a challenge, delirium can be difficult to diagnose. More recently developed delirium screening tools should be included in an advanced meta-analysis. Lastly, a network meta-analysis to evaluate healthcare interventions demonstrated the relative effectiveness of all interventions and effectively ranked the interventions even in the absence of direct comparisons.

Therefore, this study aimed to evaluate the screening accuracy of different assessment tools for mechanically ventilated patients by using a network meta-analysis method, and to rank different methods of assessment using the superiority index.

## 2 Methods and analysis

### 2.1 Registration

This protocol has been registered with the International Prospective Register of Systematic Reviews. The registration number is CRD42020153618. This systematic review protocol will follow the guidelines of Preferred Reporting Items for Systematic Reviews and MetaAnalyses (PRISMA-P).^[25]^

### 2.2 Eligibility criteria

We will include studies that met the following criteria: population limited to ICU mechanically ventilated patients; index tests that included at least 1 delirium assessment tool for diagnosed patients (e.g., CAM-ICU, ICDSC), which was compared with the reference standards (Diagnostic and Statistical Manual of Mental Disorders). sufficient information to calculate the true positive (TP), false positive (FP), true negative (TN), and false negative (FN) values; and cohort or cross-sectional designs. We did not limit the language or year of publication. We will exclude editorials, commentaries, as well as pilot, case report, and duplicated studies.

### 2.3 Search methods for identifying the studies

#### 2.3.1 Electronic sources

PubMed, PsycINFO, EMBASE, and the Cochrane Library will be searched from the study's inception to July 2020. The search strategies were developed by QZ and guided by XMY, who is an experienced evidence-based medicine researcher. The search terms were “delirium,” “acute confusion,” “diagnosis,” “sensitivity,” and “specificity.” The references of relevant systematic reviews and meta-analyses will also be searched to identify potential studies.

#### 2.3.2 Study records

EndNote X9 will be used to manage the initial search records; after removing duplicate records, the remaining records will be imported to Rayyan a free mobile app and web for systematic reviews.^[26]^ Two reviewers (YZ and QZ) will independently screen the titles and abstracts of all identified records. We will download the texts of the potential records to review them for inclusion further. Disagreements will be resolved by discussion or through consultation with a third reviewer (XY). Study selection is summarized in a Preferred Reporting Items for Systematic Reviews and Meta-Analyses flow diagram.

#### 2.3.3 Data extraction and management

Four reviewers (QZ, YZ, ZG, and ZW) will extract data from a predesigned data extraction form using Microsoft Excel 2019 (Microsoft, Redmond, WA, www.Microsoft.Com). We will collect data including their study characteristics (e.g., year of publication, surname of the first author, country where the research was conducted, reference standard, index tests used), patient characteristics (sample size, male/female, mean age, diagnostic method used, duration of the interventions) and outcomes (TP, FP, FN, TN). Conflicts will be resolved by consensus or consultation with a third reviewer (XY).

### 2.4 Quality evaluation

Applying the standards adapted from the Quality Assessment of Diagnostic Accuracy Studies 2 (QUADAS-2),^[27]^ the bias risk for each study will be graded by two reviewers(YJL and QZ) as low, moderate or high independently. This method involves four fields: selection of patients, index tests, reference standards, and flow and timing. Conflicts will be settled by negotiation. Unified results will be solved by consulting a third reviewer (XMY).

### 2.5 Statistical analysis

#### 2.5.1 Meta-analysis

A pairwise meta-analysis will be performed to calculate the pooled sensitivity (SEN), specificity (SPE), negative likelihood ratio, positive likelihood ratio, and diagnostic odds ratio using a bivariate mixed-effects regression model in MetaDiSC ver 1.4 (Unit of Clinical Biostatistics Team of the Ramón y Cajal Hospital, Madrid, Spain). Results will be reported with a 95% confidence interval. We will evaluate the heterogeneity between studies using the inconsistency index (I^2^ test; the values of 25%, 50%, and 75% I^2^ represented low, moderate, and high statistical heterogeneity, respectively,) and the Q value.^[29]^

We will use STATA 15.1 (Stata Corporation, College Station, TX) with the program “midas” to investigate publication bias. Subgroup and meta-regression analyses will be planned to further explore potential sources of heterogeneity. A priori variables that were selected as potential sources of heterogeneity were study design, reference standard, funding, and study quality.

#### 2.5.2 Quality of evidence

We will rate the evidence as “high,” “moderate,” “low,” or “very low” in a conclusive table using the Grading of Recommendations Assessment, Development and Evaluation profiler 3.2.^[30]^

## 3 Discussion

The early detection of delirium in mechanically ventilated patients is of great significance. A valid screening method will help patients reduce the duration of mechanical ventilation, lower the rate of hospital-acquired complications, and improve the quality of life after discharge. The study is the first meta-analysis to assess the accuracy of different screening methods for delirium in mechanially ventilated patients. We hope that our research will contribute to clinicians and public decision making.

## Author Contributions

YZ and QZ are cofirst authors. XY planned and designed the current study. QZ, YZ, ZG, and ZW will extract the data. QZ and YZ will perform the data analysis and initial interpretation. All authors will revise critically for important intellectual content and approve the final version to be submitted.
